# Lactate Metabolism and Lactylation Modification: New Opportunities and Challenges in Cardiovascular Disease

**DOI:** 10.1002/mco2.70269

**Published:** 2025-07-01

**Authors:** Mengyang Song, Bin Liu, Haiou Wang, Wei Sun

**Affiliations:** ^1^ Department of Cardiology The Second Hospital of Jilin University Changchun China

**Keywords:** cardiovascular disease, cellular metabolism, immunotherapy, lactylation, lactic acid

## Abstract

Traditionally regarded as a metabolic waste product, lactic acid is now recognized as a crucial molecule in energy metabolism and signal transduction. It regulates various biological processes, including intracellular inflammation and immune responses. Notably, a novel epigenetic modification, lactylation, which is directly linked to lactic acid, was first identified by Professor Zhao Yingming's team in 2019. Lactylation influences gene expression and is associated with inflammation, cancer, and ischemic disease. Despite its potential significance, the precise mechanisms by which lactylation regulates gene expression remain unclear, and its implications in cardiovascular diseases are not yet fully understood. This review elucidates the relationship between lactic acid metabolism and cellular functions, highlighting its significant biological roles. This review provides a comprehensive analysis of the mechanistic role of lactylation in disease progression. It synthesizes research findings and emerging trends concerning lactic acid production and lactylation in the context of cardiovascular diseases. We explored potential therapeutic agents targeting lactylation and identified prospective treatment targets, offering novel insights into and directions for intervention strategies related to lactic acid production and lactylation. Ultimately, this review aims to pave the way for new therapeutic and research avenues in cardiovascular diseases.

## Graphical abstract

Lactate, beyond a metabolic byproduct, regulates immunity and inflammation, linked to cardiovascular diseases (CVD) via metabolic reprogramming. Lactylation, a lactate‐driven epigenetic mark, modulates gene expression in fibrosis, lipid disorders, and CVD progression. Targeting lactylation (via GLUT1, HDACs, or CAR‐T) and combining metabolic/immune therapies offer novel strategies to treat CVD, addressing unmet needs in current drug resistance.

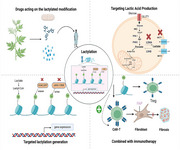

## 1 Introduction

Cardiovascular disease (CVD) is the leading cause of global mortality, imposing significant economic strain and escalating societal burdens [1, 2]. The cardiovascular system is critically dependent on the energy supply, and alterations in energy metabolic pathways can profoundly influence cardiac function [3]. Key energy metabolic pathways, including mitochondrial oxidation, glycolysis, fatty acid, and amino acid metabolism, play pivotal roles in the pathophysiological mechanisms underlying CVD development. Consequently, CVD is increasingly recognized as a disorder intrinsically linked to energy metabolism [4, 5, 6, 7, 8, 9]. The modulation of energy metabolism pathways holds substantial promise for controlling the onset and progression of CVDs, offering significant potential for treating cardiac metabolic disorders [10]. However, current pharmacological interventions for CVDs have notable limitations. For instance, while statins effectively inhibit cholesterol production and mitigate the progression of coronary heart disease, a substantial proportion of patients exhibit treatment resistance [11], and proprotein convertase subtilisin‐kexin type 9 inhibitors must be combined for synchronous treatment. Therefore, there is an urgent need for new therapeutic targets and drugs to intervene in the occurrence of cardiac metabolic diseases.

Concurrently, advancements in analytical chemistry have propelled the field of metabolomics, highlighting the synergistic interactions between small‐molecule metabolites, genes, and proteins in both physiological and pathological contexts [12]. Metabolomics facilitates the rapid analysis of metabolite profiles in complex biological matrices, and its application has significantly advanced our understanding of the pathogenesis of cardiovascular metabolic diseases. This approach is expected to yield novel diagnostic and prognostic tools and identify new therapeutic targets [13].

Lactylation is a novel epigenetic modification discovered in recent years using metabolomics techniques [14]. Lactylation is regulated by intracellular lactate metabolism and is involved in the regulation of energy metabolism, gene expression, and immune responses, thereby integrating metabolic pathways with genetic modifications [15, 16]. In CVDs, lactylation plays a critical role in pathological processes, such as inflammation, angiogenesis, dysregulation of lipid metabolism, and fibrosis. Its involvement in the pathogenesis of conditions such as atherosclerosis, myocardial infarction (MI), cardiomyopathy, pulmonary arterial hypertension (PAH), and heart failure (HF) underscores its potential as a therapeutic target [17]. Currently, relevant studies on targeted lactate therapy for cancer have entered the clinical research stage [18, 19]; however, the means of treating CVDs through targeted lactate and lactylation have not yet been systematically explored and applied.

This review elucidates the molecular mechanisms of lactic acid metabolism and lactylation and delineates their roles in the progression and pathophysiology of CVDs. This highlights the burgeoning interest in developing drugs targeting these pathways and synthesizing current research on therapeutic strategies for CVDs. Furthermore, this review explores lactylation as a prospective therapeutic target and provides theoretical and experimental insights that may inform the development of novel treatment modalities.

## 2 Lactic Acid: Turning Waste Into Treasure

### 2.1 Lactate Metabolism

Lactic acid (2‐hydroxypropanoic acid) can be classified into two enantiomeric forms, l‐lactic acid and d‐lactic acid, based on their distinct effects on polarized light [20]. Additionally, in nature, lactic acid can exist as a racemic mixture of both enantiomers, known as dl‐lactic acid [21]. In biological systems, l‐lactic acid is the most prevalent form that plays a significant role in regulating various physiological and pathological processes [22]. The synthesis of lactic acid in vivo primarily originates from two sources: the conversion of pyruvate, the end product of glycolysis catalyzed by lactate dehydrogenase (LDHA), and glutaminolysis, which is considered a secondary source of lactate production in tumors [23]. Under hypoxic conditions, the glycolytic pathway generates two pyruvate molecules accompanied by the production of adenosine triphosphate (ATP) and nicotinamide adenine dinucleotide (NADH). This pathway involves 9–10 biochemical steps and is regulated by three key enzymes, hexokinase 2, phosphofructokinase 1, and low‐activity pyruvate kinase M2 (PKM2), the rate‐limiting steps in glycolysis. Subsequently, NADH and pyruvate are reduced to lactate in the cytoplasm. Consequently, under this pathway, each glucose molecule yields two ATP molecules and two lactate molecules without consuming oxygen [24, 25].

Early studies posited that lactate is produced exclusively through anaerobic glycolysis under hypoxic conditions. However, Otto Warburg challenged this conventional view by demonstrating that tumor tissues can generate lactate from glucose even in the presence of ample oxygen, a phenomenon now referred to as the Warburg effect [26, 27]. The Warburg effect is not confined to tumor tissues; it is also observed in rapidly proliferating mammalian cells, such as pluripotent stem cells, immune cells, and endothelial cells (ECs). This metabolic reprogramming is likely because oxidative phosphorylation provides a relatively constant supply of ATP. In contrast, the Warburg effect generates ATP significantly faster, satisfying rapidly proliferating cells’ heightened energy demands. Consequently, the Warburg effect is regarded as a “hallmark of rapid proliferation” [28, 29]. The Warburg effect has been identified in various pathophysiological states of the cardiovascular system, including atrial fibrillation, PAH, and HF [30].

Lactate transport across the cellular membrane is mediated by transmembrane proteins known as monocarboxylate transporters (MCTs) [31]. MCTs belong to the solute carrier 16 (SLC16) transporter family, which comprises 14 isoforms (MCT1–14, SLC16A1–14), as well as two members of the sodium‐dependent MCT family (SMCT1/2 and SLC5A8/12). These transporters are critical in nutrient transport, cellular metabolism, and pH regulation [32]. Among these, MCT1–4 have been extensively studied and are well‐characterized as proton‐dependent transporters primarily responsible for the transport of glycolysis‐derived metabolites (e.g., lactate and pyruvate) and ketone bodies (e.g., acetoacetate and β‐hydroxybutyrate) [32]. As classical H+/lactate symporters, the direction of lactate transport by MCTs is determined by the concentration gradients of protons and monocarboxylate ions. MCT1 is the most ubiquitously expressed isoform and primarily facilitates lactate and pyruvate uptake. In contrast, MCT4 is induced by hypoxia and redox signaling and is predominantly involved in the efficient export of lactate from glycolytic cells. MCT2 exhibits functional similarities to MCT1 but is expressed at lower levels, whereas MCT3 is exclusively found in the retinal pigment epithelium and functions similarly to MCT4. The functional activity of MCT1–4 is maintained by its association with glycosylated ancillary proteins, specifically basigin (CD147) or embigin. MCT1, MCT3, and MCT4 preferentially bind basigin, while MCT2 predominantly interacts with embigin [33].

Lactate can be translocated between cells via MCTs. The current scientific consensus suggests that lactate, generated through cell glycolysis, can be distributed throughout the body and utilized as an energy substrate by various tissues. Lactate dehydrogenase B (LDHB) converts lactate into pyruvate, which subsequently enters the tricarboxylic acid (TCA) cycle and serves as a primary carbon and energy source. Alternatively, in the liver, lactate can be converted to glucose through gluconeogenesis, known as the Cori cycle, and stored as glycogen, demonstrating its role as an energy intermediate [34, 35]. Cai et al. [36] revealed a novel pathway in which lactate can directly enter the mitochondrial matrix and activate the electron transport chain to generate ATP, bypassing the conventional metabolic conversion to pyruvate. This process inhibits glycolysis while simultaneously enhancing pyruvate oxidation and lactate utilization. This finding further highlights the crucial role of lactate as a nutrient in energy provision.

### 2.2 Metabolic Pathways Regulated by Lactate

Since its discovery in 1780, lactate has been regarded as a metabolic by‐product under hypoxic conditions, with the perception that it is detrimental to organisms. After the discovery of the intercellular lactate shuttle in 1980, the role of lactate in metabolic processes gained proper recognition [37]. Lactate is indispensable for various physiological cellular functions and participates in crucial cellular processes, including energy metabolism, signal transduction, and epigenetic modification. Lactate plays a significant role in inflammation, tumorigenesis, neurological disorders, and CVDs [22].

In addition to its involvement in cellular metabolism, lactate participates in several critical cellular processes. Research has demonstrated that accumulated lactate can form an inhibitory complex with zinc at the active site of the cysteine protease SENP1, rapidly stabilizing SUMOylation of APC4 (small ubiquitin‐like modifier). This process facilitates the binding of the anaphase‐promoting complex/cyclosome (APC/C) to its transient binding partner UBE2C. The remodeling of the APC/C by lactate enhances the degradation of cyclin B1 and securin, thereby regulating mitotic exit and controlling cell cycle progression and proliferation [38].

Furthermore, lactate accumulated under hypoxic conditions protects NDRG3 protein from degradation via the prolyl hydroxylase domain 2 (PHD2)/von Hippel–Lindau‐dependent pathway, thereby increasing NDRG3 protein levels. Accumulated NDRG3 binds to c‐Raf, mediating hypoxia‐induced activation of the Raf–extracellular signal‐regulated kinase (ERK) pathway, which promotes angiogenesis and cell growth during hypoxia, thereby enhancing cellular adaptation to hypoxic stress [39]. Regardless of hypoxic or normoxic conditions, elevated lactate levels stimulate angiogenesis through the VEGF/VEGFR2 signaling pathway, facilitating wound healing under oxygen‐stimulated conditions [40, 41]. Additionally, in the hypoxia‐inducible factor 1α (HIF‐1α)‐dependent angiogenesis pathway, lactate stabilizes HIF‐1α and upregulates VEGF expression, a process independent of oxygen availability [42].

During tumorigenesis and progression, lactate modulates endothelial phenotype, cell migration, and tube formation through the NF‐κB/IL‐8 pathway, thereby promoting tumor vascular morphogenesis and perfusion [43]. Furthermore, elevated lactate levels contribute to the immunosuppression of the tumor microenvironment (TME). For instance, lactate can impair the effector function of cytotoxic T cells by interfering with the activation of the p38 and JNK/c‐Jun signaling pathways, leading to the inhibition of IFN‐γ production. Additionally, lactate maintains the IL‐23‐dependent secretion of IL‐17 in monocytes/macrophages, polarizing the immune response toward a TH17 phenotype. This results in an increased population of TH17 cells in the tumor environment, accompanied by the upregulation of inflammatory and proangiogenic factors mediated by IL‐17, thereby promoting tumorigenesis [33, 44]. Moreover, lactate drives the HIF‐1‐dependent transcription of genes encoding VEGF and the arginine‐metabolizing enzyme arginase 1, polarizing tumor‐associated macrophages toward an M2 phenotype and facilitating the immune evasion of tumor cells [33].

During intercellular signaling, lactate primarily functions as a signaling molecule by interacting with the G protein‐coupled receptor, GPR81. Although GPR81 is predominantly expressed in adipocytes, it is also present in various healthy cells, including the liver, kidneys, and heart [45]. In adipocytes, insulin‐dependent glucose uptake leads to an increased local lactate release. Lactate acts in an autocrine or paracrine manner and couples with GPR81 to inhibit adenylate cyclase via Gi‐dependent mechanisms, thereby suppressing lipolysis and mediating the insulin‐induced inhibition of adipocyte lipolysis [46]. This mechanism may explain energy metabolism patterns in the heart. Under normal conditions, the majority (95%) of myocardial ATP production is derived from the oxidation of free fatty acids, with only 5% from glucose metabolism and minimal contributions from other substrates such as lactate, ketones, and amino acids [47]. However, under stress conditions, such as ischemic coronary artery disease, HF, and shock, where the energy supply is insufficient, or utilization is impaired, the heart reduces its reliance on fatty acids. Lactate becomes the primary and preferred energy source, enhancing the rate of myocardial energy production to meet cardiac energy demands [30, 48, 49]. Wallenius et al. [50] extended the functional role of GPR81 to the cardiovascular system, demonstrating that GPR81 not only inhibits lipolysis and improves insulin resistance but also promotes endothelin release, leading to renal vasoconstriction and hypertension. This effect may be part of a systemic defense mechanism under elevated lactate levels, ensuring adequate oxygen supply to vital organs during hypoxia. Additionally, during inflammatory responses, the lactate receptor GPR81 in colonic dendritic cells and macrophages inhibits the differentiation of proinflammatory Th1/Th17 cells while increasing the production of IL‐10 by regulatory T cells (Tregs), thereby suppressing colonic inflammation and restoring homeostasis [51]. In the TME, lactate contributes to tumor proliferation, migration, and immune evasion through GPR81. GPR81 can promote angiogenesis by upregulating the PI3K/Akt/cAMP pathway and enhancing DNA repair by increasing the expression of DNA repair proteins, such as BRCA1, nibrin, and DNA–PKcs. Furthermore, GPR81 activation is involved in downstream Gi‐ and PKC–ERK signal transduction and is associated with enhanced DNA repair and chemoresistance [42]. Therefore, the lactate/GPR81 axis plays a critical role in tumorigenesis and progression and represents a promising therapeutic target for cancer treatment.

## 3 Lactoylation Modification: A Latecomer to Epigenetics

Epigenetic modifications represent a form of gene regulation that alters phenotypic traits without changing the DNA sequence, is influenced by environmental factors, and is inherited stably across generations. Among the three major epigenetic mechanisms—DNA methylation, histone modification, and RNA interference—posttranslational modifications (PTMs) of histones, including methylation, acetylation, ubiquitination, and phosphorylation, play a pivotal role [52, 53]. Lactylation has recently emerged as a novel modification pathway that has attracted considerable attention. Briefly, lactylation involves adding a lactyl group (La) to lysine residues on histone or nonhistone protein tails, which are predominantly localized in gene promoter regions. The introduction of a hydroxyl group enhances hydrogen bonding, facilitating the recruitment of chromatin remodelers, transcription factors, and histone modifiers, thereby influencing gene transcription [54, 55]. Moreover, the intracellular lactate concentration largely determines the extent of lysine lactylation (Kla). Glycolytic inhibitors reduce lactate production and subsequently decrease Kla levels, whereas mitochondrial inhibitors and cellular hypoxia increase Kla levels by elevating lactate levels, thereby modulating gene expression. Thus, lactylation serves as a bridge that links energy metabolism to gene expression [14].

Histone lactylation was first identified using liquid chromatography–tandem mass spectrometry [14]. Wan et al. [56] discovered a characteristic cyclic ammonium ion (Cyclm) as a specific fragment ion for Kla, enabling more precise identification of lactylation sites. Lactylated proteins are widely distributed in both the nucleus and cytoplasm. This approach revealed that lactylation occurs extensively in human histones and nonhistone proteins and directly regulates gene expression. Furthermore, proteomic analyses have identified lactylation sites in diverse species, including humans, mice, protozoa, plants, and microorganisms [57], demonstrating its widespread presence in nature. However, the functional roles of these lactylation sites remain largely unknown and require further investigation.

**FIGURE 1:**
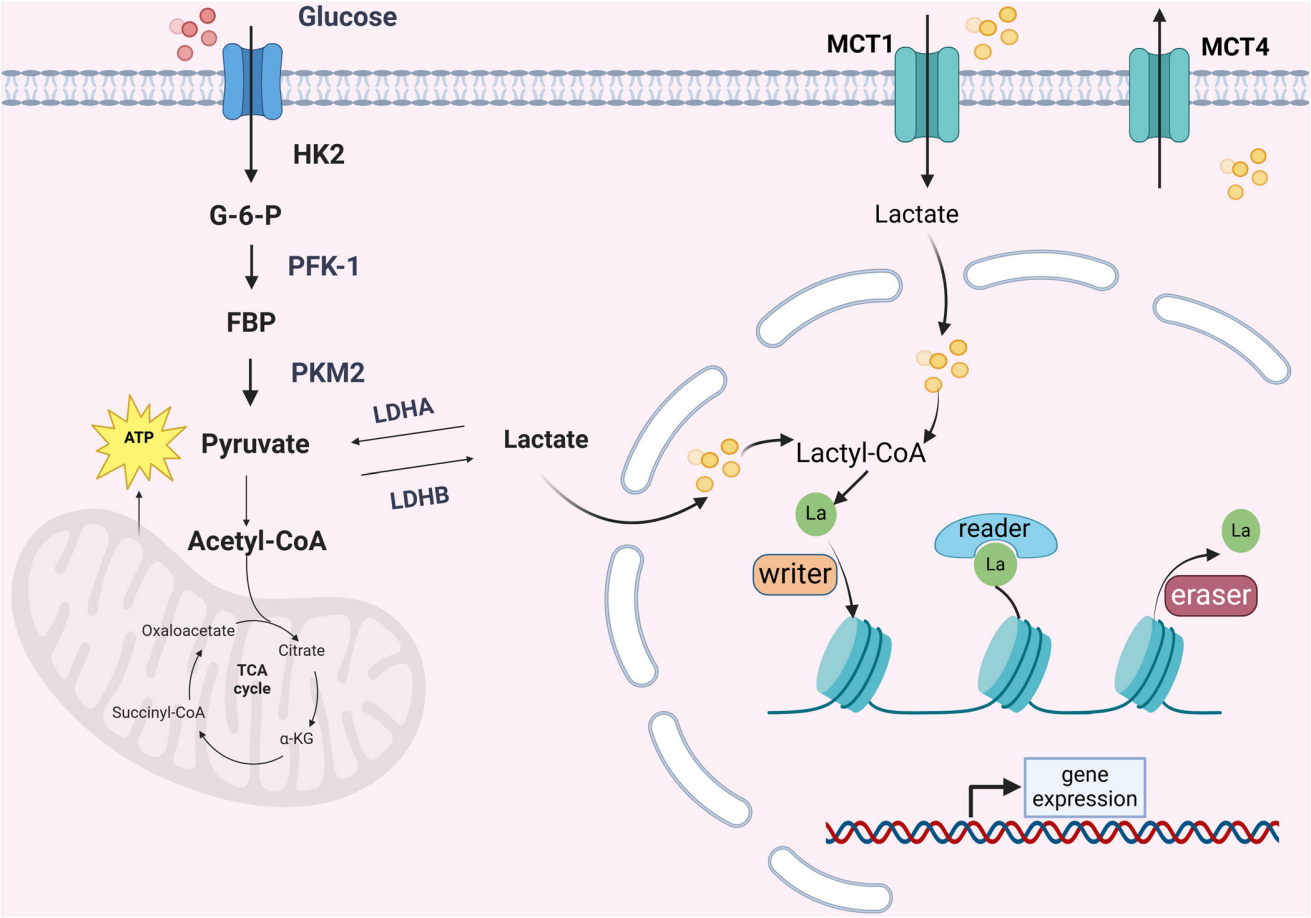
Lactate metabolism and lactylation. Glucose undergoes glycolysis to generate pyruvate, which is converted to lactate by lactate dehydrogenase A (LDHA). Lactate is transported across membranes via monocarboxylate transporters (MCT1/MCT4) and can be further metabolized into lactyl‐CoA. Lactyl‐CoA serves as a substrate for protein lactylation, a posttranslational modification regulated by “writers” (enzymes adding lactyl groups), “erasers” (enzymes removing lactyl groups), and “readers” (proteins recognizing lactylation marks). Lactylation modulates gene expression, potentially linking metabolic states to epigenetic regulation. FBP, fructose‐1,6‐bisphosphatase; MCT1/MCT4, monocarboxylate transporter 1/4; HK2, hexokinase 2; G‐6‐P, glucose‐6‐phosphate; PFK‐1, phosphofructokinase‐1; PKM2. pyruvate kinase M2; LDHA/LDHB, lactate dehydrogenase A/B; TCA, tricarboxylic acid cycle; La, lactyl group.

Based on the generation mechanism, lactylation can be classified into enzymatic and nonenzymatic types. The nonenzymatic pathway involves transferring lactyl groups from lactoylglutathione (LGSH) to protein lysine residues. LGSH is formed through rapidly conjugating methylglyoxal, a byproduct of glycolysis, with glutathione by glyoxalase 1 [58]. In contrast, the enzymatic pathway is a reversible modification regulated by three classes of proteins: “writers” (catalyzing Kla), “erasers” (removing Kla), and “readers” (recognizing Kla) [57, 59]. Professor Zhao Yingming identified p300 as a potential histone Kla writer that catalyzes the addition of lactyl groups from lactyl‐CoA to specific lysine sites. However, its intracellular mechanism remains incompletely understood [14]. In HF research, p300 was shown to catalyze lactyl group transfer at K1897 of α‐myosin heavy chain (α‐MHC) [60]. The homolog of p300, CREB‐binding protein C (CBP), increases the level of Kla in high‐mobility group box‐1 (HMGB1) [61], whereas YiaC functions as an acetyltransferase in prokaryotes [62]. HBO1 acts as an acetyltransferase, catalyzing H3K9la to mediate gene transcription [63]. Although “erasers” control lactylation without persistently affecting gene expression, class I histone deacetylases (HDAC1‐3) and SIRT1–3 proteins are potent de‐lactylases of histone lysine [64, 65]. The “reader” effector proteins specifically recognize Kla production to influence downstream signaling pathways and initiate various biological events [66]. Therefore, lactylation is a dynamic process requiring writers and erasers to regulate gene expression in vivo organically (Figure 1).

## 4 Lactic Acid Metabolism in CVDs

Recent studies have demonstrated that lactate accumulation in the cardiovascular system, resulting from various ischemic and hypoxic conditions, serves as a prognostic indicator for patient survival, particularly in critical conditions such as HF and cardiogenic shock secondary to MI [67, 68, 69, 70]. Experimental evidence from animal models has revealed a paradoxical protective role of lactate in the early phase of ischemia–reperfusion (I/R) injury. Specifically, continuous sodium lactate infusion during the resuscitation phase after cardiac arrest enhances neurological and cardiovascular recovery, potentially through mechanisms involving energy utilization and mitochondrial function [71]. However, persistent lactate accumulation is strongly associated with multiorgan dysfunction due to severe hypoperfusion, ultimately leading to poor clinical outcomes and increased mortality.

### 4.1 Lactic Acid Metabolism in Ischemic Diseases

#### 4.1.1 Lactic Acid Metabolism in Ischemic Heart Disease

A Mendelian randomization study demonstrated a significant association between elevated lactate levels and increased risk of coronary atherosclerosis and peripheral vascular disease [72]. Clinical studies have consistently shown that lactate levels are closely associated with prognosis [67, 73, 74]. Specifically, increased lactate levels are associated with higher in‐hospital mortality and short‐term mortality (30‐day and 180‐day) in patients with acute MI. In contrast, an improved 24‐h lactate clearance rate predicted reduced mortality risk [75, 76]. In carotid atherosclerosis, blood lactate concentration strongly correlates with plaque burden independent of other cardiovascular risk factors. Researchers have hypothesized that elevated lactate levels, reflecting mitochondrial dysfunction, may contribute to the development of atherosclerosis through increased production of reactive oxygen species (ROS), leading to enhanced LDL oxidation, endothelial dysfunction, apoptosis, and vascular smooth muscle proliferation [77]. However, Sun et al. demonstrated that lactate can activate GPR81 in atherosclerotic regions exposed to oscillatory shear stress, reducing oxidative stress and suppressing inflammatory factor expression. Additionally, lactate inhibits the secretion of vascular cell adhesion molecule‐1 and E‐selectin, potentially preventing monocyte adhesion to the endothelium and attenuating atherosclerosis. These effects are primarily mediated by the upregulation of Kruppel‐like factor 2 expression via the ERK 5 pathway [78]. These contradictory findings suggest that a causal relationship between elevated lactate levels and atherosclerosis warrants further investigation. Lactate may function as a double‐edged sword in atherosclerosis pathogenesis; while contributing to endothelial dysfunction, it may play a crucial role in inhibiting monocyte chemotaxis and macrophage aggregation, differentially influencing various stages of atherosclerosis development.

Lactate exerts protective effects in a porcine myocardial I/R injury model. The ischemic region exhibited significantly higher lactate concentration, lactate dehydrogenase (LDH) activity, and MCTs (MCT1 and MCT4) expression than nonischemic areas. Elevated lactate levels may facilitate ischemic myocardial repair by promoting the proliferation of synthetic vascular smooth muscle cells (vSMCs). Global proteomic analysis revealed that exogenous lactate supplementation in human‐induced pluripotent stem cell‐derived vSMCs (hiPSC–vSMCs) upregulated components of the fibrotic and injury repair pathways, including Yes‐associated protein (Yap) of the Hippo signaling pathway, mammalian target of rapamycin (mTOR), and AMP‐activated protein kinase (AMPK). These findings suggested that lactate enhanced the expression of synthetic markers and promoted the proliferation and migration of hiPSC–vSMCs, thereby contributing to myocardial injury repair [79].

#### 4.1.2 Lactic Acid Metabolism in Ischemic Encephalopathy

Proteomic and metabolomic analyses have revealed similar energy metabolism patterns in the hearts and brains of mice, exhibiting high sensitivity to hypoxia and preferentially utilizing lactate as an energy source, even in the presence of glucose [80]. Under ischemic and hypoxic conditions with glucose deprivation, glycogen is converted into lactate, which serves as the primary energy substrate for the nervous system, thereby mitigating the detrimental effects of glucose deprivation and providing neuroprotective effects [81]. Supporting this, Geiseler et al. [82] demonstrated that l‐lactate administration in a distal middle cerebral artery occlusion mouse model of stroke resulted in reduced infarct volume and increased capillary density compared with controls, an effect mediated by the lactate receptor hydroxycarboxylic acid receptor 1 (HCAR1, previously known as GPR81). However, conflicting evidence from Shen et al. [83] suggests that GPR81 inhibition may confer neuroprotection following cerebral ischemia by reversing ischemia‐induced apoptosis and ERK signaling. These paradoxical findings indicate a potential dual role of GPR81 and suggest that lactate and GPR81 may exert their effects through multiple pathways during cerebral ischemic injury. Furthermore, Shen et al. hypothesized that lactate might have concentration‐dependent effects on ischemic brain injury. Low concentrations (approximately 1–3 mmol/L) may not compensate for energy deficits during ischemia [84, 85] and could induce neuronal damage via GPR81. In contrast, higher concentrations may provide neuroprotection through ATP production, although this requires further experimental validation.

In a piglet model of cerebral I/R, researchers observed an initial increase, followed by a decrease in lactate levels, with peak concentrations occurring approximately 2–6 h posthypoxic–ischemic injury. Concurrently, the expression of MCT4, primarily localized in astrocytes for lactate efflux, and MCT2, mainly expressed in neurons for lactate uptake, change dynamically in response to lactate fluctuations. This lactate shuttling between astrocytes and neurons ensures an adequate energy supply to the nervous system [86].

Emerging evidence suggests that lactate exerts neuroprotective effects against cerebral ischemia via multiple molecular mechanisms. Recent studies have shown that lactate activates the two‐pore domain potassium channel TREK1 in a concentration‐dependent manner by interacting with histidine 328 in the carboxyl‐terminal domain of the channel. This interaction enhances TREK1 activity by reducing its prolonged closed state, potentially contributing to neuroprotection via effective potassium clearance, spatial buffering, and volume regulation [87].

### 4.2 Lactic Acid Metabolism in HF

Lactate acts as a protective agent in acute HF (AHF) progression, with exogenous lactate supplementation demonstrating therapeutic potential through anti‐inflammatory and antihypertrophic effects, thereby delaying AHF onset [88, 89]. However, in patients with established AHF, elevated lactate levels are frequently observed, even in the absence of clinical hypoperfusion‐induced hypoxia, correlating with multiorgan dysfunction (involving the liver, heart, and kidneys) and increased mortality [69]. Further investigations have revealed that lactate production and accumulation in patients with HF are predominantly determined by mixed venous oxygen saturation (reflecting tissue oxygen metabolism and utilization), heart rate, and systemic vascular resistance rather than hypoxemia or respiratory failure, elucidating the primary sources of lactate generation in HF [90].

Hypertension‐mediated cardiac hypertrophy is a critical pathological process in chronic pressure overload‐induced HF. LDHA and its metabolic product lactate play essential roles in stimulating cardiomyocyte hypertrophy and maintaining normal cardiac contractile function under pressure overload conditions. This process is mediated through the upregulation of the N‐myc downstream‐regulated gene family (NDRG), increased c‐Raf phosphorylation, and activation of ERK pathways [91]. Notably, Cluntun et al. [92] revealed that the inhibition of MCT4‐mediated lactate efflux in cardiomyocytes redirects glycolytic carbon flux toward mitochondrial pyruvate oxidation, reversing the hypertrophic phenotype and potentially offering a novel therapeutic approach for chronic HF management.

### 4.3 Lactic Acid Metabolism in Pulmonary Hypertension

A metabolomics study conducted in a Chinese population revealed significantly higher serum lactate concentrations in patients with pulmonary hypertension (PH) than in healthy controls [93], with hyperlactatemia demonstrating a strong correlation with an increased mortality risk in patients with PH [94]. The pathological hallmark of PH is the obstructive remodeling of pulmonary arterioles, primarily manifested through the progressive proliferation of pulmonary artery smooth muscle cells (PASMCs) and excessive accumulation of extracellular matrix (ECM) components, including collagen [95, 96]. Mechanistic studies have demonstrated that lactate, generated via LDHA, promotes PASMC proliferation and migration by activating the Akt signaling pathway, thereby contributing to pulmonary vascular remodeling in PH. Genetic knockdown of LDHA attenuates hypoxia‐induced lactate accumulation in murine lungs, suppresses Akt signaling activation, and ameliorates hypoxia‐induced vascular remodeling and right ventricular dysfunction, suggesting its potential role as a therapeutic target in PH pathogenesis [97]. Furthermore, increased expression of 6‐phosphofructo‐2‐kinase/fructose‐2,6‐bisphosphatase 3 (PFKFB3) is observed in the pulmonary arteries of patients with PAH and rodent models. Upregulated PFKFB3 enhances glycolysis and lactate production, leading to an increased ERK1/2 phosphorylation and subsequent calpain activation, ultimately promoting collagen synthesis and PASMC proliferation in PH. Notably, PFKFB3 silencing attenuates hypoxia‐induced PH and vascular remodeling, highlighting the crucial role of glycolysis and lactate metabolism in PH progression [98] (Table 1).

**TABLE 1:** Lactate in cardiovascular disease.

Disease	Modeling	Regulatory mechanism pathways	Effect	References
Ischemic heart disease	Crowds	ROS↑ → LDL oxidation↑ → endothelial dysfunction, enhanced apoptosis, increased vascular smooth muscle proliferation	Damage	[77]
	HUVECs, human U937 monocytes	GPR81↑ → ERK5/KLF2↑ → ①ROS↓, ②IL‐6, IL‐8, MCP‐1↓, ③VCAM‐1, E‐selectin↓	Protect	[78]
	Pigs, hiPSC–vSMC	Yap↑, mTOR↑, AMPK↑	Protect	[79]
Ischemic encephalopathy	Mice with permanent occlusion of the left distal medial cerebral artery (dMCA)	HCAR1 (GPR81)	Protect	[82]
	N2A cells, mice with middle cerebral artery occlusion (MCAO)	HCAR1 (GPR81)	Damage	[83]
	Cerebral ischemia–reperfusion in piglets	MCT4 (astrocytes), MCT2 (neuronal cells)↑	Protect	[86]
	Hippocampal CA1sr astrocytes and human embryonic kidney 293 cell line (HEK293) cells	TREK1 (H328)↑	Protect	[87]
Heart failure	Mice	NDRG↑ → c‐Raf↑ → ERK↑	Protect	[91]
	Mice	MCT4↓ → mitochondrial pyruvate metabolism↑ → cardiac hypertrophy↓	Protect	[92]
Pulmonary hypertension	Mice	LDHA↓ → lactate↓ → Akt↓ → pulmonary vascular remodeling↓	Protect	[97]
	Mouse, rat primary human PASMCs	PFKFB3↑ → lactate↑ → p‐ERK1/2↑ → calpain↑	Damage	[98]

## 5 Lactylation Modification in CVD

### 5.1 Atherosclerosis

Atherosclerosis is a chronic inflammatory disease characterized by macrophage accumulation and phenotypic switching in the arterial wall, which plays a pivotal role in determining atherosclerotic plaque fate [99]. Macrophages are classified into two phenotypes: classically activated proinflammatory (M1) and alternatively activated anti‐inflammatory (M2) macrophages [100]. M1 macrophages are associated with inflammation activation and atherosclerosis progression, whereas M2 macrophages are closely linked to inflammation resolution and atherosclerotic regression [101, 102]. Consequently, reversing atherosclerosis requires a phenotypic switch from M1 to M2 macrophages. This phenotypic transition is accompanied by metabolic reprogramming, characterized by a shift from the glycolysis‐dependent M1 phenotype to the oxidative phosphorylation and intact TCA cycle‐dependent M2 phenotype [103, 104]. Concurrently with energy metabolism transformation, lactylation has emerged as a crucial regulatory mechanism in macrophage polarization from the M1 to M2 state. Research has demonstrated that lactate progressively activates macrophage lactylation of histone H3 during inflammatory responses at lysine 18 (H3K18la) 16–24 h postinflammatory stimulation, inducing M2‐like characteristics. This process drives the synchronous upregulation of the expression of the homeostatic gene arginase 1 expression, highlighting the essential role of lactylation in late‐phase inflammatory repair [54].

Lactylation plays a crucial role in the regulation of macrophage‐mediated inflammation [105]. The activation of Sox family proteins is a critical factor in the transdifferentiation of vSMCs into macrophage‐like cells. Specifically, lactylation of sex‐determining region Y‐related HMG‐box gene 10 (Sox10) depends on phosphorylation at serine residue S24 and is essential for maintaining Sox10 activity. Under the regulation of tumor necrosis factor α (TNF‐α) and PI3K/AKT signaling, lactylated Sox10 promotes pyroptosis, vascular inflammation, and intimal hyperplasia, contributing to vascular proliferation and atherosclerotic complications. Conversely, Sox10 silencing significantly inhibits these pathological processes [106].

MCT4 is highly expressed in macrophages within atherosclerotic plaques in both humans and high‐fat diet‐fed animals. MCT4 deficiency in macrophages leads to intracellular lactate accumulation, which stimulates p300 expression and enhances histone H3 lysine 18 lactylation (H3K18la) activation. This results in the increased enrichment of anti‐inflammatory genes (e.g., IL‐10) and TCA cycle genes (e.g., PDHA1), reduced lipid uptake during macrophage activation, and promotion of macrophage‐mediated repair and resolution of inflammation. Furthermore, MCT4 deficiency ameliorates atherosclerosis by modulating inflammatory macrophage metabolic reprogramming and mitochondrial function. This was evidenced by reduced glycolytic capacity and increased oxygen consumption rate in MCT4‐deficient foam cells (FCs), accompanied by restored ATP levels. Additionally, MCT4 deficiency reversed the altered expression of TCA cycle genes (PDHA1, IDH2, SDHA, and FH) and glycolytic genes (HK2 and LDHA) in FCs [107].

Sensible and moderate exercise prevents CVD, and exercise is often accompanied by elevated lactate. Studies have shown that exercise training and exogenous lactate administration promote the lactylation of methylated CpG‐binding protein 2 (Mecp2) lysine (Mecp2k271la). Mecp2k271la is enriched in the promoter region of the epiregulator protein (Ereg). It represses the expression of Ereg by binding to its chromatin. It exerts antiatherosclerotic effects by regulating the phosphorylation of the epidermal growth factor receptor, thereby inhibiting the activity of the mitogen‐activated protein kinase (MAPK) signaling pathway. In an animal model of atherosclerosis, elevated Mecp2k271la further reduced the expression of adhesion molecules, such as vascular cell adhesion molecule 1 and intercellular adhesion molecule 1, monocyte chemotactic protein 1, and the expression of the inflammatory factors interleukin (IL)‐1β and IL‐6 by inhibiting the Ereg/MAPK pathway. At the same time, the endothelial nitric oxide synthase level in mouse aortic tissue increased, which is related to the inhibition of the adhesion and migration of ECs to macrophages, plays an anti‐inflammatory role, and promotes the regression of atherosclerosis. This study further confirms the potential therapeutic targets of exercise‐modified lactylation in atherosclerotic heart diseases [108].

### 5.2 Ischemia‐Related Diseases

#### 5.2.1 Acute MI

Emerging evidence highlights the complex dual role of lactate metabolism in cardiac remodeling following MI. Pathologically elevated lactate levels induce Snail1 lactylation through MCT‐dependent signaling. As a key transcriptional regulator of the TGF‐β pathway, lactylated Snail1 enhances Tgfb1/Smad2 signaling transduction, upregulates *TGF‐β* expression, and promotes endothelial‐to‐mesenchymal transition (EndoMT), ultimately exacerbating myocardial fibrosis and cardiac dysfunction [109]. Interventions targeting lactate reduction effectively suppress these pathological cascades, leading to improved post‐MI cardiac outcomes [109]. Furthermore, lactate—a degradation product of poly‐l‐lactic acid scaffolds—exhibits profibrotic effects by activating TGF‐β1 signaling. This process drives vascular EC transdifferentiation into mesenchymal phenotypes, characterized by downregulation of endothelial markers (PECAM‐1 and vWF), upregulation of mesenchymal markers (α‐SMA, SM22α) and profibrotic genes (collagen‐1, collagen‐3, MMP2, MMP9), and enhanced cellular contractility, collectively contributing to in‐stent restenosis [110].

In contrast, a recent study by Wang et al. [111] revealed the beneficial role of lactylation in early‐stage post‐MI ventricular remodeling. In a murine MI model, histone H3K18 lactylation (H3K18la) in bone marrow‐derived and circulating monocytes is markedly elevated during the early remodeling phase. This modification activates reparative genes, such as *Lrg1*, *Vegf‐a*, and *IL‐10*, exerting anti‐inflammatory and proangiogenic effects while initiating remote repair programs. Mechanistically, MCT1‐mediated lactate transport is indispensable for H3K18la induction, which suppresses excessive inflammation, attenuates pathological fibrosis and adverse cardiac remodeling, and significantly improves post‐MI cardiac function [111]. These findings underscore the temporally dependent nature of lactylation in the modulation of myocardial repair.

The apparent contradictions among these studies may align with the proposed “lactylation clock” hypothesis by Zhang et al. [14]. This model posits that lactylation orchestrates protective repair mechanisms via the precise spatiotemporal regulation of gene networks during specific post‐MI phases. However, cumulative effects across overlapping time windows may shift lactylation toward promoting pathological remodeling. This hypothesis emphasizes the critical need to investigate lactylation dynamics along the temporal axes, necessitating advanced spatiotemporal models to dissect context‐dependent regulatory mechanisms.

#### 5.2.2 Myocardial I/R Injury

In both I/R‐induced mouse models and oxygen‐glucose deprivation/reperfusion‐treated neonatal rat cardiomyocytes, lactate levels were significantly elevated, accompanied by an increased expression of glycolysis‐related genes (Aldoa, Pfkm, and Pkm2). In I/R 3‐week mice, H3K18la expression was markedly upregulated by lactate induction. Further investigation revealed that lactylation deposits in the YTHDF2 promoter region transcriptionally regulated the expression of the RNA N6‐methyladenosine (m6A)‐binding protein YTHDF2. Overexpression of YTHDF2 in conjunction with G3BP1 (an essential RNA‐binding protein for stress granule assembly) inhibited cardiomyocyte size and exacerbated apoptosis. In vivo experiments demonstrated that YTHDF2 knockdown reduced infarct size following acute I/R injury, prevented long‐term cardiac dysfunction, and attenuated cardiac fibrosis, thereby mitigating pathological remodeling and exerting cardioprotective effects. Notably, exercise training reduces lactate production and subsequently inhibits YTHDF2 by decreasing lactylation, highlighting its therapeutic potential in myocardial I/R injury [112].

In contrast, Yu et al. [113] reported that H3 lactylation (H3K56la) was suppressed in cardiomyocytes during postischemic reperfusion, along with the downregulation of aerobic glycolysis‐related genes (including HK‐II and LDHA) and HSPA12A, a member of the heat shock protein family. Gain‐ and loss‐of‐function studies revealed that HSPA12A, during hypoxia/reoxygenation stress, enhanced Smurf1‐mediated Hif1α protein stability, thereby regulating aerobic glycolysis activity, promoting lactate production, and maintaining H3 lactylation (H3K56la) to support cardiomyocyte survival and attenuate myocardial I/R injury. Similarly, postreperfusion lactate elevation upregulates Serpina3k lactylation at lysine 351, thereby increasing its stability and expression. Further investigation demonstrated that I/R‐stimulated fibroblasts secreted Serpina3k/SERPINA3, which acts on cardiomyocytes in a paracrine manner. This cardioprotective mechanism involves WNT pathway inhibition and activation of the reperfusion injury salvage kinase pathway (including AKT and ERK1/2) and the survivor‐activating factor enhancement pathway, protecting cardiomyocytes from I/R‐induced apoptosis and mitigating myocardial reperfusion injury [114].

These seemingly contradictory findings suggest that the cardioprotective mechanisms of lactylation in the ischemic myocardium may depend on specific target proteins and their associated signaling pathways, potentially offering novel therapeutic targets for myocardial ischemia protection.

#### 5.2.3 Ischemic Encephalopathy

In cerebral I/R (CI/R) injury, intracellular lactate promotes pyroptosis via histone H3 lysine 18 lactylation (H3K18lac). Knockdown of LDHA significantly reduced both lactate and H3K18lac levels and decreased H3K18lac occupancy at the proximal promoter region of high‐mobility group box 1 (HMGB1), thereby suppressing HMGB1 expression and subsequently inhibiting pyroptosis in CI/R [115]. Furthermore, glycolysis inhibition and lactate production reduce the lactylation of lymphocyte cytosolic protein 1 (LCP1), an actin‐binding protein in cerebral infarction models, leading to LCP1 degradation, apoptosis suppression, and ultimately attenuation of CI progression [116]. Lactylation participates extensively in CI/R, primarily influencing biological processes such as cell projection morphogenesis, the Wnt signaling pathway, brain development, neuronal projection regeneration, and injury response. It also affects molecular functions, including protein kinase activity and energy transfer, as well as signaling pathways involving calcium ions, cGMP–PKG, and MAPK [117].

#### 5.2.4 Ischemic Fundi Disease

Neovascularization of the eye is a major cause of blindness [118]. Lactylation in the ischemic fundi promotes angiogenesis and fibrosis. Chen et al. [119] found elevated expression of fat mass and obesity‐associated protein (FTO), an m6A demethylase, in patients with diabetic retinopathy and a mouse model and that it can be demethylated through m6A–YTHDF2‐dependent *CDK2* mRNA to promote *CDK2* expression, which, in turn, acts on EC cycle progression and tip cell formation. It is also involved in neovascularization in diabetic retinopathy. Further studies have found that the disturbance of lactate homeostasis, a common feature of diabetic retinopathy, can affect FTO via lactylation, thus playing the role described above [119].

In addition, lactylation of nonhistone proteins plays a role in proliferative retinopathy, and Yin Yang‐1 (YY1), a multifunctional transcription factor that binds to specific DNA sequences in several promoters and enhancers to promote or repress transcription, is involved in the regulation of angiogenesis [120, 121, 122]. Hypoxia increases lactate levels in microglia, thereby increasing the lactylation of YY1 at lysine 183 (K183) and facilitating its binding to the FGF2 promoter in a manner regulated by the acylation writer p300. This promotes FGF2 transcription through hyperlactylation, upregulates FGF2 expression, and promotes retinal neovascularization, all of which participate in the development of proliferative retinal lesions [118].

Myopia is partly caused by increased scleral fibroblast‐to‐myofibroblast trans‐differentiation, which is facilitated by increased glycolysis in the sclera during hypoxia, upregulation of the glycolysis/lactate/histone lactation cascade due to lactate accumulation, and activation of *Notch1* transcription by H3K18la [123]. This process is consistent with myocardial fibrosis after MI and requires further investigation in myocardial fibrosis.

#### 5.2.5 Ischemic Nephropathy

Significantly elevated lactate/creatinine and renal lactate levels were found in the urine of mice with I/R injury and patients with CKD. Meanwhile, fructose‐6‐phosphate‐2‐kinase/fructose‐2,6‐bisphosphatase 3 (PFKFB3) is a key glycolytic enzyme that is significantly upregulated in renal proximal tubular cells following I/R injury in mice. Lactate accumulation after PFKFB3‐induced enhancement of renal tubular glycolytic reprogramming significantly enhanced histone H4 lysine 12 lactylation (H4K12la), which, in turn, was enriched at the promoters of NF‐κB signaling genes (e.g., *Ikbkb*, *Rela*, and *Relb*), activating their transcription and promoting an inflammatory response accompanied by the presence of key proinflammatory cytokines as well as the upregulation of chemokines *Tnf‐a*, *Il‐6*, *Il‐1β*, and *Ccl2*, which are involved in the development of renal interstitial fibrosis. The application of oxalate (a LDH inhibitor) significantly reduced IRI‐induced H4K12 lactylation; inhibited the expression of proinflammatory factors and chemokines IL‐1β, IL‐6, and Ccl2; effectively ameliorated renal fibrosis; and confirmed a new therapeutic target for fibrosis [124].

### 5.3 Heart Failure

Diabetic cardiomyopathy primarily manifests as diastolic dysfunction, which can progress to HF in severe cases [125]. Recent studies have highlighted the critical role of lactate metabolism in this pathological process. Ma et al. [126] identified lactate as an independent predictor of diastolic dysfunction in patients with type 2 diabetes mellitus (T2DM), demonstrating a significant negative correlation between elevated lactate levels and the E/A ratio, a key echocardiographic parameter of diastolic function. Mechanistically, the upregulation of MCT4 in cardiomyocyte membranes disrupts the intracellular lactate–pyruvate balance. The extruded lactate enhances macrophage inflammation by increasing H4K12 lactylation (H4K12La) levels, promoting HIF‐1α transcription, and amplifying free fatty acid‐induced inflammatory responses, ultimately exacerbating myocardial injury. Notably, MCT4 inhibition significantly attenuates this pathological cascade [126].

Further insights into the role of lactate in cardiac function have been gained from studies on protein lactylation. Zhang et al. [60] discovered that lactylation at lysine 1897 (K1897) of the α‐MHC, crucial for its interaction with titin and maintenance of sarcomere integrity, is markedly reduced in failing hearts of both mice and humans. Mice with K1897 mutations exhibited impaired α‐MHC–titin binding and compromised myosin stability, exacerbating HF. Interestingly, exogenous sodium lactate supplementation restored α‐MHC K1897 lactylation and enhanced α‐MHC–titin interaction, thereby ameliorating cardiac dysfunction. Further mechanistic investigations revealed that p300 and sirtuin 1 function as the acyltransferase and deacylase for α‐MHC lactylation, respectively. LDHA and MCT4, the key regulators of lactate metabolism, modulate lactylation levels by controlling intracellular lactate concentrations in HF [60].

### 5.4 Pulmonary Hypertension

Under hypoxic conditions, mitochondrial ROS accumulation stabilizes and enhances nuclear HIF‐1α levels in PASMCs by inhibiting HIF‐1α hydroxylation. This stabilization process triggers a metabolic shift toward glycolysis through HIF‐1α‐mediated upregulation of two key glycolytic enzymes, pyruvate dehydrogenase kinase 1 (PDK1) and PDK2, resulting in increased lactate production and subsequent histone lactylation (H3K18la, H4K5la). Mechanistic investigations revealed that this histone lactylation specifically enhances the expression of HIF‐1α target genes involved in cellular proliferation, including Bmp5, Trpc5, and Kit, promoting PASMC proliferation. Importantly, pharmacological inhibition of LDH significantly attenuated histone lactylation, suppressed PASMC proliferation, and improved vascular remodeling in a rat model of hypoxic PH [127]. These findings establish a novel link between cellular energy metabolism and vascular remodeling, providing potential therapeutic targets for PH intervention through the modulation of metabolic pathways.

### 5.5 Calcification of the Aortic Valve

Currently, there are no effective pharmacological therapies to halt the progression of calcific aortic valve disease (CAVD); surgical intervention remains the primary treatment option [128]. Emerging evidence has highlighted the crucial role of lactylation in the pathogenesis of CAVD. Comparative analyses revealed significantly elevated pan‐lactylation (Pan Kla) levels in patients with CAVD, with osteogenic medium‐induced valvular interstitial cells (VICs) demonstrating increased lactylation, particularly at the H3Kla and H3K9la sites. This enhanced H3Kla level promotes the expression of the calcification‐related gene runt‐related transcription factor 2 (Runx2), suggesting a potential mechanism for CAVD development [129].

**FIGURE 2:**
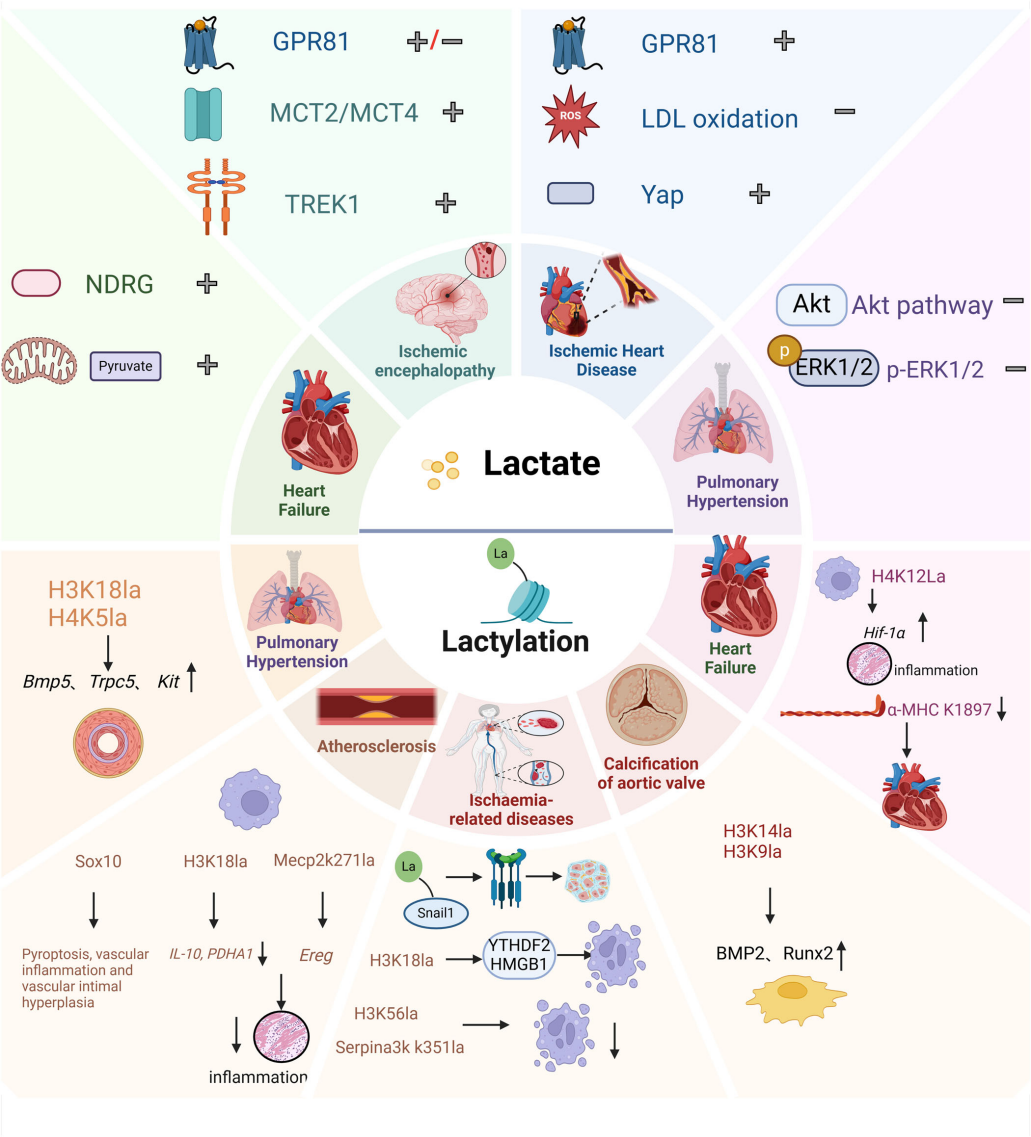
Lactate and lactylation in cardiovascular and ischemia‐related diseases. This schematic diagram illustrates the multiple roles of lactate and lactylation in cardiovascular diseases and ischemia‐related diseases. Lactate, a byproduct of glycolysis, accumulates under hypoxic conditions and is transported via monocarboxylate transporters (MCT2/MCT4). It can activate G‐protein‐coupled receptor 81 (GPR81) and regulate signaling pathways such as Akt and ERK1/2, which influence cell survival, inflammatory responses, and vascular remodeling, playing either protective or detrimental roles in the heart and ischemic diseases. Lactylation is a posttranslational modification that regulates protein function by modifying lysine residues (e.g., α‐MHC K1897la, HSK18la), thereby affecting processes such as inflammation, vascular calcification (via BMP2/Runx2), and pyroptosis, and exerting multiple effects in the pathogenesis of cardiovascular diseases. GPR81: G‐protein‐coupled receptor 81; MCT2/MCT4: monocarboxylate transporter 2/4; TREK1: TWIK‐related K+ channel 1; Yap: Yes‐associated protein; NDRG: N‐Myc downstream‐regulated gene; Akt: protein kinase B; ERK1/2: extracellular signal‐regulated kinase 1/2; Hif‐1α: hypoxia‐inducible factor‐1α; BMP2: bone morphogenetic protein 2; Runx2: runt‐related transcription factor 2; IL‐10: interleukin‐10; HMGB1: high mobility group box 1; PDHA11: pyruvate dehydrogenase alpha 1; α‐MHC K1897la: α‐myosin heavy chain lysine 1897 lactylation; HSK18la: histone H3 lysine 18 lactylation. (+) Protective mechanisms; (–) detrimental mechanisms.

Furthermore, Huang et al. [130] observed marked increases in intracellular lactylation levels (H3K14la and H3K9la) and calcification‐related proteins (Runx2 and BMP2) in human calcified aortic valve specimens. Their investigation identified lumican (LUM), a small leucine‐rich proteoglycan, and ubiquitous ECM component [131], as key regulators of this process. LUM overexpression activated inflammatory pathways and enhanced cellular glycolysis via CD44 receptor binding, leading to lactate accumulation and histone lactylation at specific H3 sites (H3K14la and H3K9la). This lactylation enhancement correlates strongly with the expression of the calcification factors BMP2 and Runx2, which promote VIC osteogenesis and aortic valve calcification. Pharmacological inhibition using 2‐deoxy‐d‐glucose and LDHA knockout significantly suppressed histone lactylation and attenuated valve calcification [130]. These findings establish LUM and its mediated lactylation as promising pharmacological targets for CAVD intervention (Figure 2) (Table 2).

**TABLE 2:** Lactylation in cardiovascular disease and ischemia‐related diseases.

Disease	Modeling	Lactated modified proteins	Regulatory mechanism pathways	Effect	References
Atherosclerosis	Carotid artery ligation in mice	SOX10	TNF‐α↑ → PI3K/AKT↑ → SOX10↑ → (i) macrophage markers C3, Cd74, and Lyz2↑; (ii) TNF‐α, IL‐1β, and IL‐6↑; (iii) adhesion molecules VCAM‐1 and MCP‐1; and the expression of IL‐1β, IL‐18, GSDMD, and pyroptosis‐associated genes including caspase‐1, and caspase‐3 expression↑	Damage	[106]
	THP‐1‐derived macrophages, foam cells (FC) High‐fat diet‐fed Apoe^KO^ mice	H3K18	*MCT4↓* *→ lactate↑* *→* *p300↑* *→* *H3K18la↑* *→* *IL‐10*, PDHA1↑	Protect	[107]
	High‐fat diet‐fed mice, mouse aortic endothelial cells (MAEC)	Mecp2	Ereg/Egfr/MAPK↓ → Vcam‐1↓, Icam‐1↓, Mcp‐1↓, IL‐1β↓, IL‐6↓, Enos↑	Protect	[108]
Acute myocardial infarction	Mouse and mouse endothelial cells	Snail1	Tgfβ/smad2↑ → EndoMT↑	Damage	[109]
	New Zealand White Rabbit, Human Umbilical Vein Endothelial Cells (HUVECs)	Lactic acid	Tgfβ↑ → EndoMT↑ → PECAM‐1, vWF↓, α‐SMA, SM22α↑, collagen‐1, collagen‐3, MMP2, MMP9↑	Damage	[110]
	Mouse myocardial infarction model	H3K18	*Lrg1*, *Vegf‐a*, and IL‐10↑	Protect	[111]
Myocardial ischemia–reperfusion injury	Mouse, neonatal rat cardiomyocyte (NRCM)	H3K18	YTHDF2↑ → G3BP1↑	Damage	[112]
	Mouse, neonatal rat cardiomyocyte (NRCM)	H3K56	HSPA12A↑ → Smurf1↑ → Hif1α↑ → lactate↑→H3K56la↑	Protect	[113]
	Mice	Serpina3k↑	WNT↓ → AKT, ERK1/2, SAFE pathway↑ → apoptosis↓	Protect	[114]
Ischemic encephalopathy	Oxygen‐glucose deprivation/reoxygenation (OGD/R)‐treated N2a cells and middle cerebral artery occlusion (MCAO)‐treated rats	H3K18	HMGB1↑	Damage	[115]
	Middle cerebral artery occlusion (MCAO)‐treated rats and oxygen‐glucose deprivation/reoxygenation (OGD/R)‐stimulated PC12 cells	LCP1	—	Damage	[116]
Ischemic fundus disease	HUVEC, mouse	FTO	CDK2↑	Damage	[119]
	OIR mouse model, human microglia clone 3 (HMC3) cells	YY1 (K183)	FGF2↑	Damage	[118]
Ischemic nephropathy	Deprivation myopia (FDM) in mice and guinea pigs	H3K18	*Notch1*↑ → FMT↑	Damage	[123]
	Mouse, primary mouse TEC cells	H4K12	NF‐κB → signaling genes (*Ikbkb*, *Rela, Relb*)↑ → *Tnf‐a*, *Il‐6*, *Il‐1β*, *Ccl2*↑	Damage	[124]
Heart failure	H9C2, primary mouse cardiomyocytes (PMCM)	H4K12	Hif‐1α↑	Damage	[126]
	Mice	α‐MHC K1897	Titin↑	Protect	[60]
Pulmonary hypertension	Rat, PASMC	Lactic acid	Bmp5, Trpc5, and Kit lactylation↑, H3K18, H4K5 lactylation↑	Damage	[127]
Calcification of the aortic valve	valve interstitial cells (VICs)	H3K, H3K9	H3Kla↑ → Runx2↑	Damage	[129]
	Mice, VICs	H3K14, H3K9↑	LUM/CD44↑ → lactate↑ → H3K14la, H3K9la↑ → BMP2, Runx2↑	Damage	[130]

## 6 Therapeutic Targeting of Lactate Metabolism and Lactylation

### 6.1 Targeting Lactate Metabolism

Current lactylation research strategies primarily focus on lactate production pathways and their biological implications. In tumor biology, lactate‐shuttling mechanisms facilitate intercellular communication between tumor cells and their microenvironment, significantly influencing tumor invasion, metastasis, and immune evasion processes [132]. The accumulation of lactate within the TME contributes to immunosuppression, thereby promoting tumor growth and progression [133]. Therapeutic interventions targeting lactate metabolism in oncology mainly involve inhibiting lactate production and blocking lactate transport. Small‐molecule inhibitors, particularly those targeting LDHA, have demonstrated promising antitumor and antimetastatic potential in preclinical studies [134]. Additionally, lactate transporter inhibitors, such as MCT1, have emerged as potential therapeutic targets for impeding lactate efflux and attenuating tumor progression [135]. Traditional Chinese medicine (TCM) has gained increasing recognition as a disease treatment. Acupuncture protects against cerebral ischemia by inhibiting glycolysis under ischemic conditions, thereby reducing lactate production and subsequent lactylation [136].

Notably, the similarities between TME mechanisms and CVD pathogenesis, particularly in cellular proliferation and inflammatory responses, suggest that these therapeutic strategies apply to cardiovascular research.

### 6.2 Targeting Lactylation Machinery

The identification and characterization of lactyltransferases, which are enzymatic mediators of lactylation, remain an active area of investigation. Targeting these enzymes offers a promising strategy for directly modulating lactylation‐dependent gene expression without altering systemic lactate levels. Several small‐molecule drugs originally developed as acetyltransferase inhibitors have demonstrated cross‐reactivity with lactylation pathways, suggesting their potential as novel therapeutic targets. Among these, A‐485, a specific p300 writer antagonist, has been shown to effectively inhibit protein lactylation (Kla) formation and improve functional recovery in ischemic stroke models [137]. Furthermore, C646, a known inhibitor of p300/CREB acetyltransferase activity [138], also exhibits lactylation‐inhibiting properties [61], providing valuable insights into the crosstalk and interplay between different epigenetic modifications and potentially opening new research directions.

Regarding lactylation erasers, particularly HDACs, several inhibitors, including trichostatin A, MS275, nicotinamide, RGFP966, and RGFP109, have been developed. Although these compounds have demonstrated efficacy in modulating inflammation during hypoxia through nonlactylation pathways [139, 140], their specific effects on delactylation processes require further investigation. Comprehensive clinical trials are necessary to evaluate the safety and efficacy of these potential therapeutic agents in lactylation‐related disorders.

### 6.3 Immunomodulatory Therapies Involving Lactate

Recent advances have highlighted the pivotal role of both the innate and adaptive immune systems in cardiometabolic diseases, with immunomodulatory interventions emerging as promising therapeutic strategies for targeting metabolic dysregulation [141]. Lactate modulates immune responses through multiple mechanisms: it increases H3K18 lactylation levels, thereby attenuating Th17 cell pathogenicity and promoting the reprogramming of proinflammatory T cells into Tregs [142]. Additionally, lactate inhibits CD8+ T cell cytokine release (including IFN‐γ, TNF‐α, and IL‐2), induces natural killer T cell apoptosis, and fosters an immunosuppressive TME [143, 144, 145]. Elevated lactate and lactylation levels also enhance Treg‐mediated immunosuppression [146, 147], which may compromise the efficacy of chimeric antigen receptor (CAR)‐T cell therapy in solid tumors [148, 149]. Pharmacological inhibition of LDHA with agents such as oxamate reduces lactate levels and suppresses lactylation, particularly the enrichment of H3K18la at the promoters of ectonucleotidases CD39 and CD73 in Tregs or macrophages, thereby attenuating the immunosuppressive TME and enhancing CAR‐T cell efficacy [150].

Similarly, redirected T‐cell immunotherapy has been successfully applied to pathological cardiac fibrosis in murine models. The adoptive transfer of CAR‐T cells targeting fibroblast activation protein significantly reduces postinjury cardiac fibrosis and improves functional recovery, marking a new era in cardiac immunotherapy [151, 152]. Given the involvement of immune cell alterations and elevated inflammatory burden in myocardial fibrosis and coronary artery disease [153, 154], the modulation of lactate and lactate levels in the cardiac microenvironment may optimize the efficacy of immunotherapeutic interventions.

Immune checkpoint proteins, critical regulators of T cell function, have revolutionized cancer treatment through immune checkpoint inhibitors. These proteins also play significant roles in the inflammatory pathways associated with coronary artery disease. Inhibition of the CD40L–CD40 and CD80/CD86–CD28 costimulatory pathways modulates T‐cell activation and prevents atherosclerosis [155, 156], albeit with increased adverse events [157], necessitating more precise therapeutic targets. Given that lactylation directly binds to CD39 and CD73 promoters in Tregs, combining lactylation modulation with immune checkpoint inhibitor therapy may yield more effective therapeutic strategies.

**FIGURE 3:**
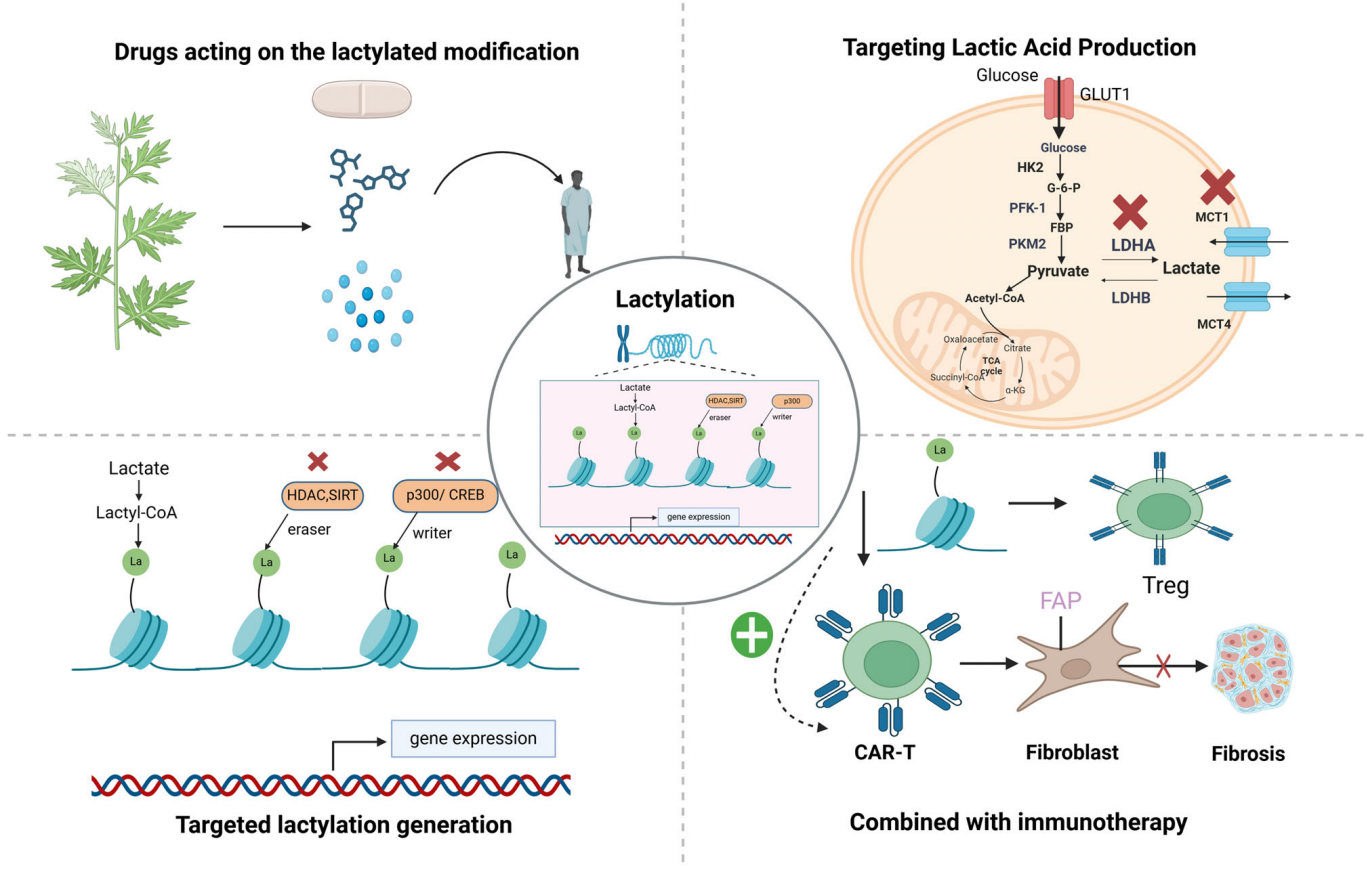
Therapeutic strategies targeting lactylation modification. This schematic outlines therapeutic strategies targeting lactate production and lactylation modification in disease treatment. Lactate, derived from glycolysis via glucose transporter 1 (GLUT1), is metabolized to lactyl‐CoA, a substrate for lactylation—a posttranslational modification regulated by “writers” (e.g., histone acetyltransferases such as p300/CREB‐binding protein (CBP) and “erasers” (e.g., histone deacetylases/sirtuins (HDACs/SIRTs). Modulating these enzymes alters lactylation levels, thereby influencing gene expression and cellular processes. Targeting lactylation in fibroblasts attenuates fibrosis, while combining lactylation modulation with immunotherapy (e.g., chimeric antigen receptor T‐cell [CAR‐T] therapy) enhances therapeutic efficacy. These approaches highlight the potential of lactylation‐centric interventions in metabolic and immune‐related pathologies. CAR‐T, chimeric antigen receptor T‐cell; CBP, CREB‐binding protein; GLUT1, glucose transporter 1; HDAC, histone deacetylase; SIRT, sirtuin.

Lactate and lactylation are promising therapeutic targets, although they are in the early stages of exploration. Currently, one clinical trial (NCT01791595) is evaluating AZD3965, an MCT1/2 inhibitor developed by AstraZeneca that suppresses cell proliferation without altering glycolytic flux [158]. Preliminary results in patients with solid tumors, diffuse large B‐cell lymphoma, or Burkitt lymphoma [159] may provide valuable insights into CVD management. The therapeutic potential of targeting lactylation in CVDs remains vast and warrants further investigation (Figure 3).

### 6.4 Pharmacological Advances

Numerous pharmacological agents can modulate lactylation pathways, influencing disease progression and therapeutic outcomes. Certain bioactive components from TCM have also demonstrated unexpected efficacy in regulating lactylation processes. This section systematically summarizes these findings to elucidate additional lactylation‐mediated mechanisms and provides valuable insights for future drug development. By integrating the established pharmacological knowledge with novel discoveries, we aim to expand the therapeutic potential of lactylation modulation in various diseases.

#### 6.4.1 Metformin

Recent studies have indicated the potential of metformin as a glucose‐lowering agent and its promising role in the treatment of inflammation‐related diseases [160, 161, 162]. In an in vivo model of inflammation in zebrafish, metformin attenuated the inflammatory response produced by oxidative stress by inhibiting neutrophil migration to the site of injury and suppressing the expression of proinflammatory cytokines (*TNF‐α*, *Il‐1β*, *Il‐6*, and *cxcl8a*). In addition, metformin inhibits lactate production by suppressing the expression of the lactate‐related genes *hdac3*, *pkma*, and *gapdh*, which further reduces histone (H3K18) lactylation, leading to a reduction in ROS production, thereby exerting anti‐inflammatory effects [163].

#### 6.4.2 Salvianolic Acid (A/B)

The salvianolic acid family (A–E) is a water‐soluble phenolic acid derived from *Salvia miltiorrhiza* with a wide range of pharmacological values [164]. Numerous studies indicate that salvianolic acid has antioxidant, anti‐inflammatory, antibacterial, antitumor, antithrombotic, and cardioprotective activities and is widely used in the prevention and treatment of cardiovascular and cerebrovascular diseases [165, 166].

Tanshinic acid A inhibits the phosphorylation and nuclear translocation of the Y105 site of pyruvate kinase muscle isoform 2 (PKM2), a key enzyme in glycolysis, which, in turn, inhibits its transcriptional activity and reduces lactate production. This results in the blockage of lactate‐dependent phosphorylation of PKR (a regulator of NLRP3 inflammatory vesicles) assembly, activation of NLRP3 inflammatory vesicles, and, consequently, the inhibition of cellular pyroptosis. Therefore, SAA inhibits EC death and alleviates diabetic atherosclerosis via the PKM2/PKR/NLRP3 inflammasome signaling pathway [167].

Tanshinic acid B (Sal B), a member of the tanshinic acid family, is the major component of water‐soluble phenolic acids in tanshin roots [164]. Recent studies have shown that Sal B has anti‐inflammatory, antiorgan damage, and antifibrotic effects in various diseases [168, 169, 170]. It was found to inhibit lactate production by downregulating the expression of LDHA in lipopolysaccharide (LPS)‐stimulated macrophages and a mouse model of hepatic injury, thus inhibiting lactylation of histone H3 lysine 18 (H3K18la), which, in turn, impedes the ability of H3K18la to bind to the LDHA, NLRP3, and IL‐1β genes, inhibits the secretion of cellular inflammatory mediators, and plays an anti‐inflammatory role in liver injury [171]. Additionally, Sal B regulates macrophage polarization in I/R hearts by interfering with macrophage glycolysis [172]. Future studies should elucidate the role of Sal B as an inhibitor of lactylation‐related pathways in cardiac diseases.

#### 6.4.3 Scopolamine

Scopolamine, a widely used inducer of neurological damage in in vivo animal models, also targets lactylation [173, 174]. Wu and Gong [174] found that scopolamine promotes osteogenic differentiation of human periodontal ligament stem cells (PDLSCs) by promoting the lactylation of RUNX2 at the K176 locus, which enhances the protein stability of RUNX2 by decreasing ubiquitination and promoting the osteogenic differentiation of PDLSC, thereby helping regenerate lost periodontal tissues and repair bone defects.

#### 6.4.4 Progesterone

Ecdysis is a progesterone‐dependent cellular differentiation process essential for establishing pregnancy [175]. Lactate produced by the endometrium plays a key role in ensuring normal ecdysis formation by establishing local histone lactylation, and progesterone can play a role in embryo implantation by promoting transcriptional activation of *Ldha* to increase lactate production and H4K12la levels, which, in turn, creates an H4K12la–Hif1α–glycolytic feedback loop to drive ecdysis [176].

#### 6.4.5 Chinese Herbal Formula Turbidifier and Fat Regulator Granules

From the perspective of the Chinese medicine theoretical system, the herbal formula “hepatotoxicity and lipotropic granules” (HTG) mainly treats dyslipidemia by regulating spleen function and promoting turbidity. HTG contains seven herbs: *Atractylodis macrocephalae*, Zhi Shi, Hepino, Shan Zha, Dan Shen, Bixi, and Hu Zhang. In vitro studies have shown that HTG increases hepatocyte glycolytic activity and lactate concentration. Further analysis of 198 proteins identified in LO2 and HepG2 cells revealed that histone H2B (K6) and H4 (K80) lactylation was upregulated and that these lactylated proteins, which are enriched in RNA processing or cellular metabolism, can play a role in improving lipid accumulation [177]. This study links lipid metabolism to gene regulation and provides a new therapeutic strategy for dyslipidemia.

#### 6.4.6 Andrographolide

Andrographolide (AGP) exerts stable anticalcification effects in vivo. Further cellular experiments revealed that AGP inhibited the expression of the calcification genes BMP2 and Runx2 by inhibiting the expression of LDHA, thereby affecting glycolysis and reducing lactate production. Conversely, it can inhibit the lactylation of lysine residues in H3 histones by altering the enzymatic activity of p300 to inhibit the expression of Runx2, which plays a role in reducing aortic valve calcification [129].

#### 6.4.7 Royal Jelly Acid

Royal jelly acid (RJA) is the major unsaturated fatty acid in naturally occurring complex royal jelly, possessing antitumor and anti‐inflammatory properties [178, 179]. During the development of hepatocellular carcinoma, RJA inhibited cancer progression by inhibiting cancer cell proliferation and migration and promoting apoptosis. Multiomics studies have shown that RJA can play an antitumor role by interfering with the glycolysis pathway. Further studies have revealed that RJA can inhibit the levels of *LDHA* and *LDHB* in HCCLM3 cells, interfere with the production of lactic acid, and inhibit the lactylation of H3 histones at H3K9la and H3K14la sites. The tumor survival curve showed that LDHA and LDHB levels were negatively correlated with survival time. This indicated that the higher the *LDHA* level, the earlier the cancer recurrence, the more invasive the cancer, and the shorter the survival time. The expression of Pan Kla was positively correlated with *LDHA* and the antiapoptotic protein BCL2 and negatively correlated with the apoptotic protein *CASP8* (caspase 8). Therefore, RJA can regulate the expression of Pan‐Kla via *LDHA*, thereby exerting antitumor effects [180]. In the near future, the mechanism of action of RJA in hepatocellular carcinoma related to lactylation regulation may provide ideas for the potential role of RJA in treating other diseases.

#### 6.4.8 Evodiamine

Evodiamine inhibits tumor growth, angiogenesis, and proliferation in prostate cancer (PCa) by affecting the lactylation pathway. An increase in lactate content in the tumor environment significantly increased the occupancy of HIF1A DNA by H3K18la in PC‐3 cells and increased the expression of HIF‐1α and HIF1α‐mediated PD‐L1 while limiting the expression of Sema3A in PCa cells and promoting angiogenesis. In contrast, silencing the expression of MCT4 reversed this effect. Conversely, evodiamine inhibits PD‐L1 transcription by restricting histone lactylation and the expression of HIF1A in PCa cells while further enhancing Sema3A transcription and inducing iron death by decreasing the expression of glutathione peroxidase 4 (GPX4), which significantly blocks lactic acid‐induced angiogenesis and thus could act as a metabolic epigenetic regulator to exert antitumor effects as an epigenetic regulator of metabolism [181].

#### 6.4.9 Dexamethasone

Asthma control relies heavily on the application of corticosteroids. Recent studies have shown that dexamethasone can exert systemic anti‐inflammatory effects in eosinophilic asthma by affecting the glycolytic process; inhibiting the expression of key glycolytic enzymes Hif‐1α, Glut1, HXKII, LDH, and PDK1 in macrophages; and inhibiting the production of lactic acid and, subsequently, lactylation. However, the underlying mechanism requires further investigation [182].

#### 6.4.10 Proanthocyanidins

Proanthocyanidins (PAs) are widely distributed in the plant kingdom as natural antioxidants with antibacterial, antitumor, antidiabetic, and neuroprotective properties [183]. More importantly, they exert cardioprotective effects by vasodilating the blood vessels and inhibiting lipid peroxidation, platelet aggregation, and capillary hyperpermeability [184, 185]. In a rat model of periodontitis and inflamed periodontal tissues of LPS‐stimulated human PDLSCs, lactylation levels were significantly reduced. In contrast, using PAs significantly increased the production of lactic acid. They restored the lactylation levels of PDLSCs, accompanied by elevated expression of the osteogenic differentiation genes ALP, RUNX2, and BMP2, thereby enhancing the osteogenic phenotype of inflamed PDLSCs. Further studies have revealed that the downstream target of lactylation may be the Wnt/β‐catenin pathway. The specific mechanism of action and lysine lactylation sites require further study [186].

#### 6.4.11 Fargesin

Fargesin, a lignan isolated from the methanolic extract of magnolia, has anti‐inflammatory, ameliorative, dyslipidemic, and insulin‐resistant effects [187, 188]. Guo et al. [189] showed that in non‐small cell lung cancer, fargesin could interfere with tumor glycolysis by targeting the key rate‐limiting enzyme PKM2, inhibiting the production of lactate, and inhibiting the lactylation of H3 histone, accompanied by the downregulated expression of proliferation‐associated proteins CDK1, CCND1, and BCL2 and upregulated expression of the apoptosis‐associated protein BAX, thereby inhibiting tumor cell proliferation and migration and exerting antitumor effects.

#### 6.4.12 Demethylzeylasteral

Demethylzeylasteral is a natural compound isolated from the entire plant or peeled woody parts of *Lei Gong Hook F*. It possesses various pharmacological properties, including anti‐inflammatory effects, modulation of estrogen metabolism, and modulation of immune system activity [190].

In liver cancer stem cells (LCSCs), lactylation was upregulated, which was closely related to tumor growth. In contrast, demethylzeylasteral inhibited lactate production in a dose‐dependent manner, mainly through the regulation of the glycolysis/glycolysis metabolic pathway, and further inhibited the lactylation of H3 histone proteins (H3K9la and H3K56la), which was also found to be downregulated by detecting the surface biomarkers CD133 and cell cycle protein cyclin D1 in LCSC. These findings suggest that lactylation inhibits tumorigenesis by suppressing LCSC proliferation and migration and promoting apoptosis [191].

#### 6.4.13 Aloe‐emodin

Aloe‐emodin, a bioactive compound derived from medicinal plants such as Aloe vera and Rheum palmatum, has been extensively studied for its cardiovascular protective effects through its anti‐inflammatory properties and ROS‐scavenging capabilities, establishing its traditional use in cardiovascular protection [192, 193, 194].

Recent mechanistic studies revealed novel molecular pathways underlying the therapeutic effects of aloe‐emodin. Specifically, aloe‐emodin stabilizes mitochondrial aspartate aminotransferase‐mediated kynurenine metabolism and interacts directly with protein disulfide isomerase P4HB. This interaction inhibits lactylation at the K311 site of P4HB, thereby reducing its lactylation and expression levels. Subsequently, this leads to the suppression of nuclear‐phosphorylated glycogen synthase kinase 3β (p‐GSK3β) transcription, inhibiting the interaction between prostaglandin G/H synthase 2 (PTGS2) and the SH3 domain of GRB2‐like protein B1 (SH3GLB1). This cascade reduces SH3GLB1‐mediated mitochondrial ROS (mitoROS) accumulation and inhibits nuclear domain‐containing protein 2 (NDP52)‐induced mitophagy. Through modulation of the PTGS2/SH3GLB1/NDP52 axis, aloe‐emodin effectively attenuates radiation‐induced myocardial fibrosis and mitigates radiation‐associated cardiac injury [195] (Table 3).

**TABLE 3:** Drugs acting on the lactylated modification.

Veterinary drug	Illnesses	Modeling	Lactate‐modified proteins	Regulatory mechanism pathways	References
Metformin	Inflammatory	Zebrafish models of inflammation	H3K18	hdac3, pkma and gapdh↓ → lactate↓ → H3K18la↓ → ROS↓	[163]
Salvianolic acid A	Diabetic atherosclerosis	HUVEC, male ApoE−/− mice	—	PKM2↓ → lactate↓ → PKR/NLRP3↓	[167]
Salvianolic acid B	Liver injury, liver fibrosis	LPS‐stimulated RAW264.7 cells, mouse model of liver injury	H3K18	LDHA↓ → lactate↓ → H3K18la↓ → NLRP3/IL‐1β↓	[171]
Black henbane	Periodontal damage	Human PDLSCs	RUNX2 K176	Enhanced RUNX2 stability	[174]
Progesterone	Metamorphosis	Ovariectomised mouse model	H4K12	*Ldha*↑ → lactate↑ → H4K12la–*Hif1α*–glycolysis feedback loop↑	[176]
Turbidity and fat regulation granules	Hypertriglyceridemia	Human hepatocyte cell lines LO2, HepG2	H2BK6, H4K80	—	[177]
Andrographolide	Calcification of the aortic valve	Mouse aortic valve calcification model, valve interstitial cells (VICs)	H3K	LDHA↓ → lactate↓ → H3Kla↓ → BMP2 and Runx2↓ p300↓ → H3Kla↓ → Runx2↓	[129]
Royal jelly acid	Hepatocellular carcinoma	HCC cell lines (Hep3B and HCCLM3), nude mouse transplantation tumor models	H3K9 and H3K14	*BCL2↓*, *CASP8↑* (Caspase 8)	[180]
Evodiamine	Prostate cancer	Human umbilical vein endothelial cells (HUVECs), human mCRPC PC‐3 (bone metastasis) and DU145 (brain metastasis) cell lines, mouse	H3K18↓	HIF1A↓ → Sema3A↑, PD‐L1↓ GPX4↓ → iron death↑	[181]
Dexamethasone	Eczema	Mouse, human macrophage cell line THP‐1	Pan Kla	Hif‐1α, Glut1, HXKII, LDH, PDK1↓ → lactate↓	[182]
Proanthocyanidin	Periodontitis	Rat, human periodontal ligament stem cells (PDLSC)	Pan Kla↑	Wnt/β‐catenin↑ → ALP, RUNX2, BMP2↑	[186]
Fargesin	Non‐small cell lung cancer	human NSCLC cell line (A549), nude mouse	Pan Kla↓	PKM2↓ → lactate↓ → CDK1, CCND1 and BCL2↓, BAX↑	[189]
Demethylzeylasteral	Hepatocellular carcinoma	LCSC in human hepatocellular carcinoma cell lines HCCLM3 and Hep3B, female nude mice	H3K9, H3K56↓	CD133, cyclin D1↓	[191]
Aloe‐emodin	Heart damage caused by radiation	Rats and H9C2 cells treated with X‐rays.	P4HB K311la↓	p‐GSK3B↓ → PTGS2/SH3GLB1/NDP52↓ → mitoROS↓ → fibrosis↓	[195]
AZD3965	Tumor	Clinical trial	MCT1/2	Cell proliferation↓	[158]

## 7 Summary and Future Development Directions

Currently, lactate serves primarily as a diagnostic and prognostic biomarker in clinical practice; however, the precise mechanisms underlying its involvement in pathological processes, such as inflammation, tumorigenesis, and immunosuppression, remain incompletely understood. The discovery of protein lactylation has opened new avenues for research, providing novel insights into protein function and establishing a crucial link between energy metabolism and gene expression, thereby bridging metabolomics and epigenetics. This breakthrough significantly enhanced our understanding of the role of lactate in pathophysiological processes.

However, several critical challenges remain in lactylation research. First, although lactate is established as the primary substrate for lactylation and its metabolic regulation through glycolysis is well characterized, nonenzymatic lactylation suggests alternative substrates. The metabolic pathways that convert lactate to lactyl‐CoA and the mechanisms governing lactyl‐CoA metabolism remain unclear. Therefore, identifying the proteins involved in lactyl‐CoA transport and synthesis and investigating the related metabolic pathways is paramount. Second, limitations in lactylation detection technologies may constrain the identification of lactylated proteins and their modification sites [196]. Recent advancements in affinity enrichment, multidimensional separation, and biological mass spectrometry have significantly improved Kla omics analysis [197]. Notably, Wang et al. [198] developed a histone tail‐based photoaffinity probe for the precise analysis of proteins interacting with histone PTMs, which, combined with proteomics, holds promise for unveiling the regulatory targets and mechanisms of PTMs. Third, although PTMs play crucial epigenetic roles by controlling protein structure and function to regulate gene expression, the extensive crosstalk between different PTMs complicates the study of Kla. The integration of high‐throughput metabolomics has emerged as a promising approach to investigate lactylation crosstalk and explore its impact on signaling pathways and cellular functions [199, 200, 201]. Finally, the specific molecular mechanisms through which lactylation regulates cellular functions and contributes to disease pathogenesis remain unclear. The lack of large‐scale clinical data models highlights the need to integrate clinical data with molecular mechanisms to understand disease progression better and inform drug development.

The development of biological materials offers exciting opportunities for targeted therapy. Combining small‐molecule compounds targeting lactate and lactylation with nanomaterials may enhance drug specificity and stability and minimize side effects. A recent study demonstrated the effectiveness of 2DG‐encapsulated poly (lactic‐co‐glycolic acid) (PLGA) nanoparticles in combining nanomaterial delivery systems with lactate inhibition, significantly reducing lactate production and improving drug‐targeting precision and concentration [202]. This approach represents a promising direction for future precision medicine research.

The field of lactylation remains largely unexplored, with lactylation modification emerging as a significant research direction and a potential therapeutic target in CVDs. Given its regulatory roles in inflammation, angiogenesis, and lipid metabolism, targeting lactylation holds substantial promise for preventing and treating CVDs. Future research should focus on developing and clinically evaluating small‐molecule compounds and novel drugs, ultimately leading to more effective therapeutic strategies.

## Author Contributions

Mengyang Song and Bin Liu contributed equally to this work. Wei Sun contributed to the conception and the design and drafting of the manuscript. Mengyang Song, Bin Liu, and Haiou Wang contributed to the data collection and manuscript drafting. All authors approved the final version of the manuscript for submission.

## Ethics Statement

The authors have nothing to report.

## Conflicts of Interest

The authors declare no conflicts of interest.

## Data Availability

The authors have nothing to report.
